# Rare MSI-H hepatoid adenocarcinoma of the colon with BRAF V600E mutation achieving long-term disease-free survival after adjuvant envafolimab: a case report

**DOI:** 10.3389/fimmu.2025.1698410

**Published:** 2025-11-19

**Authors:** Shaoqing Fan, Qingyu Meng, Zeming Zhao, Wenbo Niu

**Affiliations:** 1 Department of General Surgery, The Fourth Hospital of Hebei Medical University, Shijiazhuang, Hebei, China; 2 Department of General Surgery, The Second Hospital of Hebei Medical University, Shijiazhuang, Hebei, China

**Keywords:** colorectal neoplasms, DNA mismatch repair, adenocarcinoma, hepatoid, BRAF V600E mutation

## Abstract

**Case presentation:**

Microsatellite instability-high (MSI-H) or mismatch repair-deficient (dMMR) colorectal cancer (CRC) is characterized by high tumor mutational burden and strong immunogenicity, making it responsive to immune checkpoint inhibitors. Hepatoid adenocarcinoma (HAC) of the colon is an exceptionally rare and aggressive subtype, often resistant to conventional chemotherapy. We report a 77-year-old woman who presented with progressive anemia and a right-sided colonic mass. She underwent laparoscopic radical right hemicolectomy, and pathology revealed hepatoid features with vascular and neural invasion. Immunohistochemistry showed loss of MLH1, PMS2, and MSH6, confirming dMMR status, and MSI testing indicated MSI-H. BRAF V600E mutation was identified, and germline testing excluded Lynch syndrome. Given her age and potential chemotherapy toxicity, she received eight cycles of adjuvant envafolimab (200 mg every 3 weeks). Over 38 months of follow-up, she remained disease-free without experiencing any grade ≥2 immune-related adverse events.

**Conclusion:**

This case illustrates that adjuvant PD-1 blockade can be effective and well-tolerated in elderly patients with rare MSI-H CRC subtypes, including BRAF-mutated HAC. Comprehensive molecular profiling can help guide personalized immunotherapy decisions. Further studies are needed to confirm long-term benefits, optimize treatment duration and dosing, and identify predictive biomarkers for high-risk CRC.

## Introduction

Colorectal cancer (CRC) remains one of the leading causes of cancer incidence and mortality worldwide. Microsatellite instability-high (MSI-H) occurs in approximately 15–25% of CRC cases, while Lynch syndrome, a hereditary mismatch repair (dMMR) deficiency, accounts for only 2–3% of CRCs (1–3). It is important to note that MSI-H is not solely attributable to Lynch syndrome; approximately 70–80% of MSI-H CRCs arise from somatic alterations, such as MLH1 promoter methylation.

Recent advances in immunotherapy have shown remarkable efficacy in MSI-H/dMMR CRC. These tumors typically exhibit a high tumor mutational burden and abundant neoantigen expression, making them readily recognizable by the immune system and highly responsive to immune checkpoint inhibitors (ICIs) such as envafolimab (4).

Hepatoid adenocarcinoma (HAC) of the colon is an extremely rare CRC subtype, accounting for less than 1% of cases. Among reported HACs, colonic involvement is exceedingly uncommon (5). HAC morphologically resembles hepatocellular carcinoma, is characterized by alpha-fetoprotein (AFP) production, and exhibits aggressive behavior with resistance to conventional chemotherapy, early liver metastasis, and poor prognosis (6, 7). Most patients are diagnosed at an advanced stage, with approximately two-thirds succumbing within one year (8).

Here, we report a case of sporadic MSI-H HAC of the colon in an elderly patient who achieved long-term disease-free survival following adjuvant envafolimab therapy, without experiencing any grade ≥2 immune-related adverse events (irAEs). This case highlights the importance of molecular profiling in elderly CRC patients and provides clinical evidence supporting personalized immunotherapy strategies.

## Case presentation

### Clinical assessment

A 77-year-old woman with no family history of cancer presented with progressive fatigue and pallor over the course of one month. Laboratory testing revealed iron-deficiency anemia (hemoglobin 71 g/L). Physical examination identified a firm, poorly mobile mass in the right lower abdomen, measuring approximately 6 × 5 cm. Serum tumor markers were within normal limits, with carcinoembryonic antigen (CEA) at 2.1 ng/mL and carbohydrate antigen 19-9 (CA 19-9) at 15 ng/mL.

**Figure 1:**
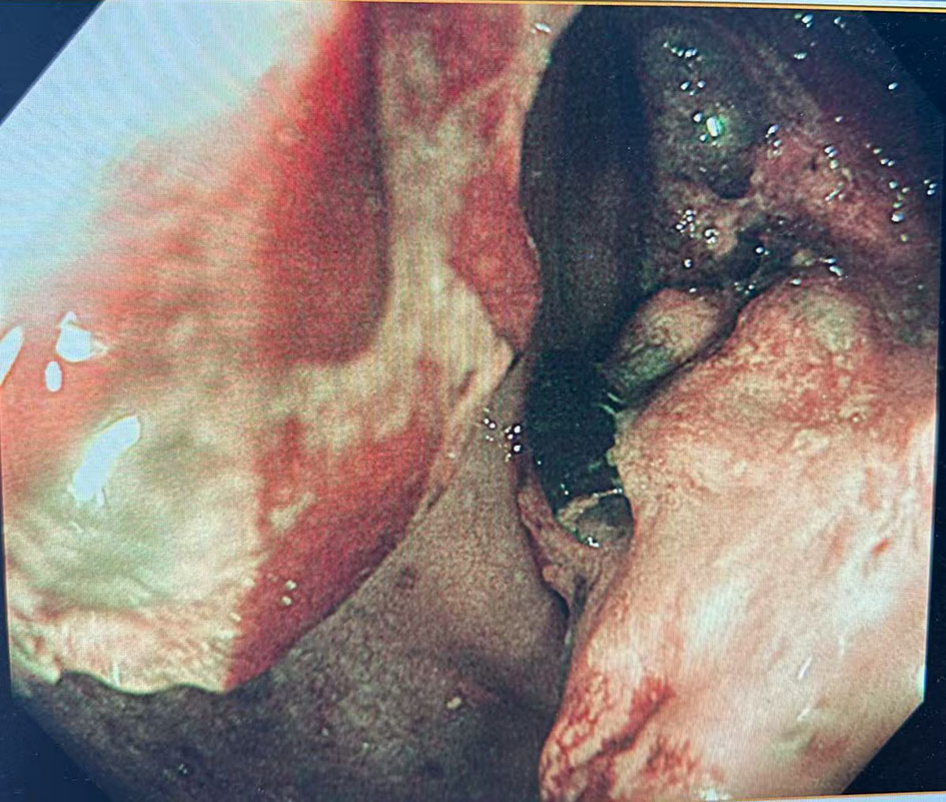
Colonoscopic showing an ulcerative stricture in the ascending colon.

**Figure 2:**
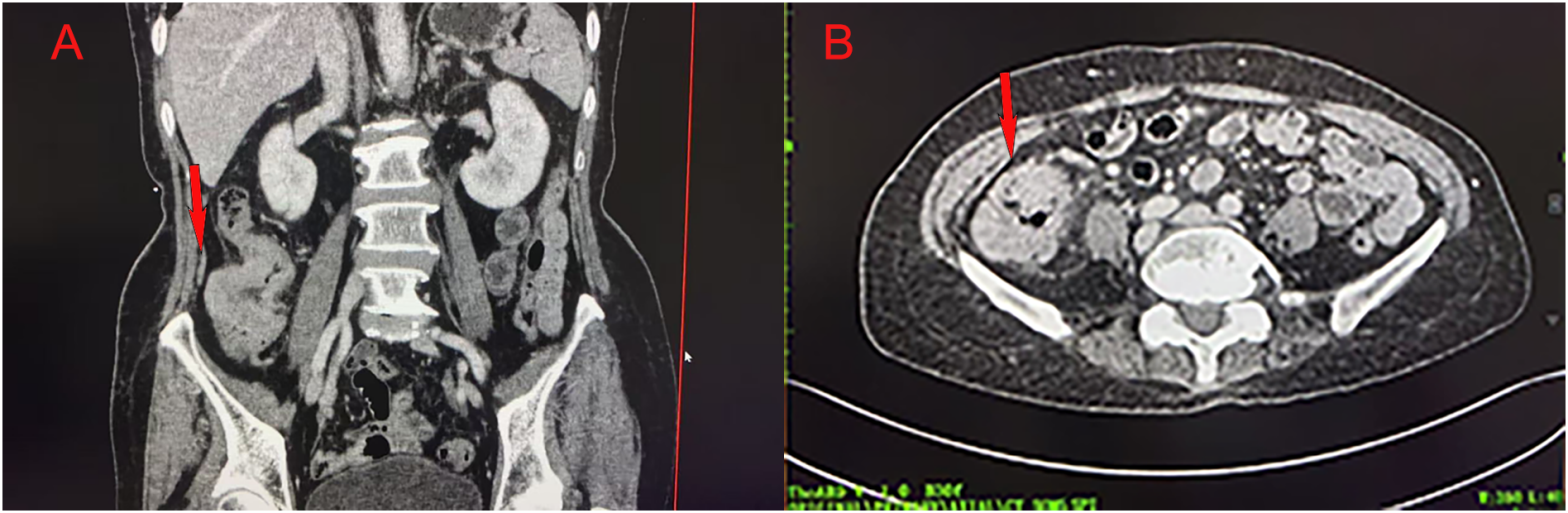
Abdominal CT showing an ascending colon mass in coronal **(A)** and axial **(B)** views.

Colonoscopy demonstrated an ulcerative stricture in the ascending colon, and histopathological examination of the biopsy confirmed poorly differentiated adenocarcinoma (**Figure 1**). Imaging studies, including thoracoabdominal computed tomography (CT), revealed an ascending colon mass with mildly enlarged regional lymph nodes (**Figure 2**). The patient had no significant comorbidities, and preoperative cardiopulmonary evaluation confirmed suitability for surgery.

### Surgical and pathological findings

**Figure 3:**
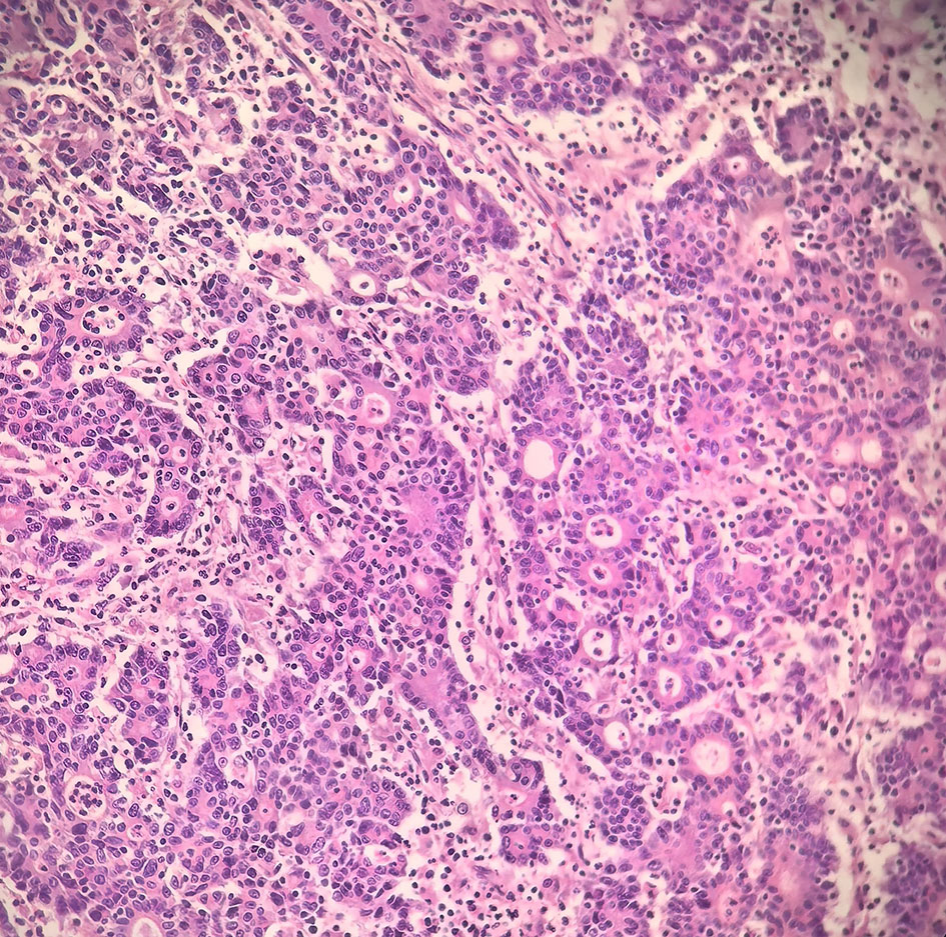
Postoperative pathology showing hepatoid adenocarcinoma differentiation with poor differentiation (Hematoxylin–eosin staining, ×200).

The patient underwent a laparoscopic radical right hemicolectomy. Histopathological examination revealed a hepatoid adenocarcinoma (HAC) (**Figure 3**) with vascular and perineural invasion, staged as pT4N0M0 (stage IIB). Immunohistochemistry demonstrated positivity for hepatocellular differentiation markers, including AFP, GPC3, and HepPar-1, as well as colorectal lineage markers, including CEA, CDX2, CK20, and SATB2. Markers CK7 and CD56 were negative, excluding other primary origins. APC (–) and TP53 (+) were observed, consistent with the molecular profile frequently reported in hepatoid adenocarcinoma. The Ki-67 proliferation index was elevated at 80%, indicating high proliferative activity (**Table 1**).

**Table 1:** Immunohistochemical (IHC) characterization of the tumor.

Category	Marker	Result
Hepatic Differentiation	AFP	+
	GPC3	+
	HepPar-1	+
Colorectal Origin	CEA	+
	CDX2	+
	CK20	+
	SATB2	+
Exclusion Markers	CK7	-
	CD56	-
Molecular Markers	APC	–
	TP53	+
Proliferation index	Ki-67	80%

### Molecular pathology

**Figure 4:**
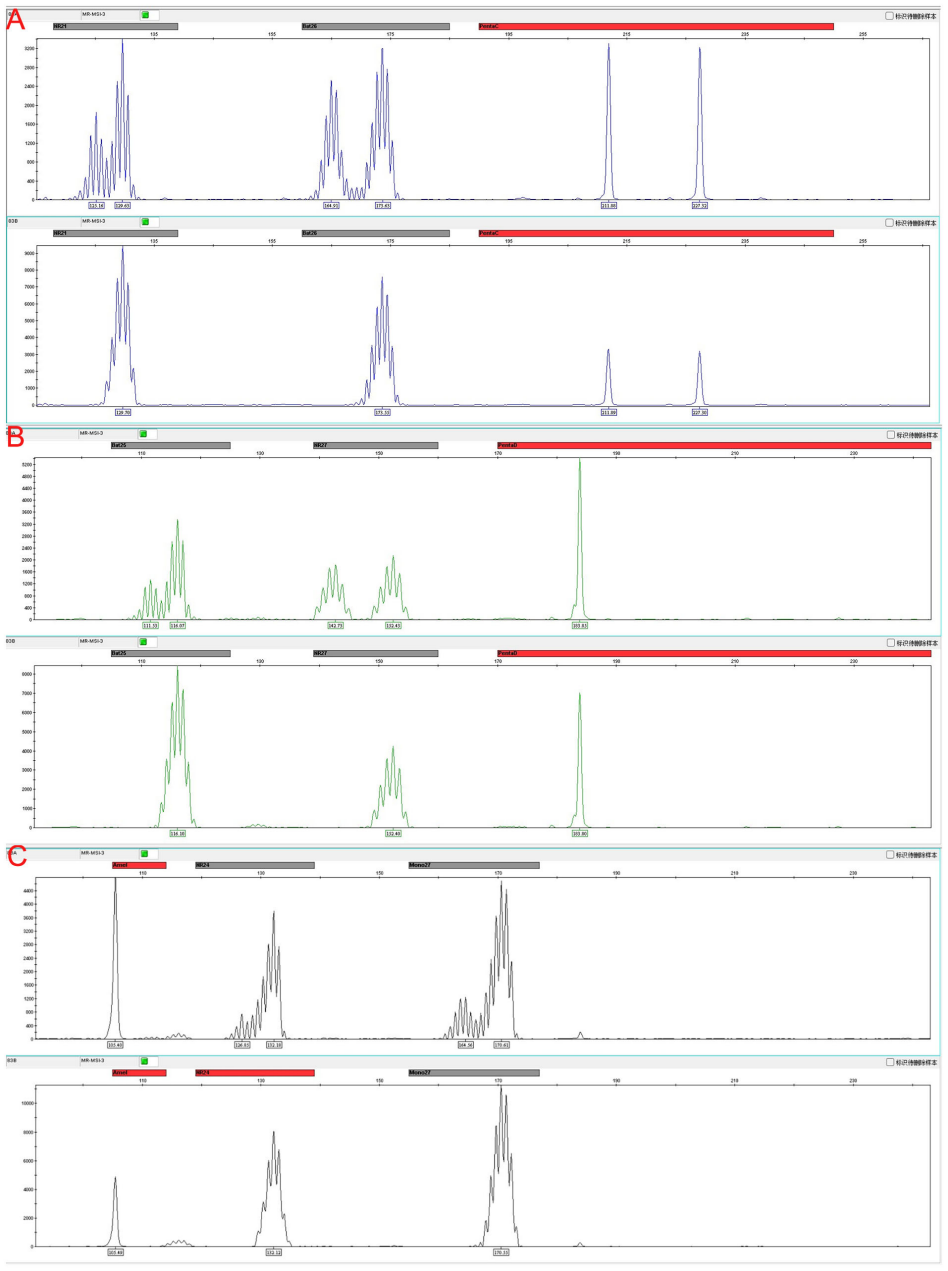
Microsatellite instability (MSI) analysis at six loci with matched tumor, adjacent non-tumor tissue, and peripheral blood DNA as controls. **(A)** Instability detected at BAT-26 and NR-21 loci. **(B)** Instability detected at BAT-25 and NR-27 loci. **(C)** Instability detected at NR-24 and MONO-27 loci. Representative electropherograms show allele shifts in tumor samples compared with stable patterns in adjacent non-tumor tissue and peripheral blood, confirming MSI-high status.

Immunohistochemistry revealed loss of mismatch repair (MMR) proteins MLH1, MSH6, and PMS2, with retained expression of MSH2, consistent with dMMR status (**Table 2**). Multiplex fluorescent PCR followed by capillary electrophoresis of six microsatellite loci (BAT-25, BAT-26, NR-21, NR-24, NR-27, MONO-27) (**Figure 4**)demonstrated instability at all sites, confirming MSI-H status (**Table 3**).

**Table 2:** Mismatch repair(MMR) protein expression profile in tumor tissue.

MMR Protein	Expression Status
MLH1	**-**
MSH2	**+**
MSH6	**-**
PMS2	**-**

**Table 3:** Microsatellite instability (MSI) status at commonly tested loci.

Microsatellite marker	Status
BAT-25	Unstable
BAT-26	Unstable
NR-21	Unstable
NR-24	Unstable
NR-27	Unstable
MONO-27	Unstable

Genetic testing showed wild-type KRAS and NRAS, but a BRAF V600E mutation was identified. To exclude Lynch syndrome, germline testing via Sanger sequencing was performed in two adult offspring, revealing no pathogenic MMR gene variants.

### Treatment and follow-up

The patient presented with three high-risk pathological features (T4 stage, vascular invasion, and perineural invasion). Due to her age (>75 years) and potential chemotherapy-related toxicity, standard platinum-based combination chemotherapy was deemed unsuitable, and single-agent fluoropyrimidine therapy has limited efficacy in MSI-H CRC (9). After informed consent from the patient and her family and multidisciplinary team evaluation, adjuvant immunotherapy with envafolimab (200 mg every 3 weeks) was administered for eight cycles.

During 38 months of follow-up, serial imaging, including abdominal CT and liver MRI, showed no evidence of local recurrence or distant metastasis. The patient did not experience any grade ≥2 immune-related adverse events (irAEs). Only mild, grade 1 nausea and occasional diarrhea were reported. No vomiting, hypothyroidism, immune-related pneumonitis, or other severe irAEs occurred. Routine hematological and biochemical monitoring, including complete blood count, liver and renal function, thyroid function, and cardiac enzymes, remained within normal limits, indicating that adjuvant PD-1 therapy was well tolerated.

## Discussion

Hepatoid adenocarcinoma (HAC) is a rare and highly aggressive extrahepatic adenocarcinoma, first reported in a gastric tumor (10). HAC frequently presents with elevated serum AFP and characteristic hepatocellular morphology, and is associated with poor prognosis. It commonly expresses hepatocellular markers such as AFP, HepPar-1, and GPC3 (11). Colorectal-specific markers, including CDX2, CK20, and SATB2, help confirm the primary origin, distinguishing HAC from metastatic hepatocellular carcinoma or other malignancies. In this case, immunohistochemistry and molecular profiling confirmed both hepatoid differentiation and colorectal origin.

MSI-H/dMMR CRC is characterized by high tumor mutational burden (TMB) and abundant neoantigen expression, often accompanied by increased tumor-infiltrating lymphocytes (TILs) and upregulation of immune checkpoint molecules, which collectively enhance sensitivity to immune checkpoint inhibitors (ICIs) (12, 13). Large clinical trials such as KEYNOTE-177 and CheckMate-142 have demonstrated significant and durable responses to PD-1 blockade in metastatic MSI-H/dMMR CRC, now recommended as first-line therapy in international guidelines (4, 14). In this patient, immunohistochemistry revealed intact MSH2 expression but loss of MLH1, MSH6, and PMS2, indicating that the dMMR phenotype was primarily related to the MLH1/PMS2 axis. MLH1 and PMS2 form a stable heterodimer essential for DNA mismatch repair; loss of MLH1 typically results in concurrent PMS2 deficiency. This pattern usually reflects somatic MLH1 mutations or promoter hypermethylation rather than hereditary MMR deficiency (15, 16).

All six analyzed microsatellite loci were unstable, confirming a classical MSI-H phenotype with high genomic instability. This likely contributed to increased TMB and abundant neoantigens, fostering a T cell–inflamed tumor microenvironment (13). Tumors with instability at only one or two loci generally exhibit lower immunogenicity and reduced responsiveness to ICIs (17). Thus, multi-locus MSI assessment provided a molecular rationale for the durable response to adjuvant envafolimab in this rare HAC.

MLH1 promoter hypermethylation and BRAF V600E mutation are common molecular features of sporadic MSI-H CRC. BRAF V600E frequently indicates MLH1 promoter hypermethylation leading to somatic dMMR rather than hereditary MLH1 mutations, supporting a diagnosis of sporadic MSI-H CRC. In this patient, the MSI-H phenotype was driven primarily by MLH1 promoter hypermethylation and somatic BRAF V600E mutation, rather than germline MMR defects (18–20). While BRAF V600E is typically associated with poor prognosis and increased tumor aggressiveness (13), evidence suggests that PD-1 blockade can partially overcome its negative prognostic impact in MSI-H/dMMR CRC (21). The patient’s durable disease-free survival following adjuvant envafolimab supports the potential benefit of ICIs in BRAF-mutant MSI-H CRC.

Management of elderly CRC patients requires balancing efficacy with treatment tolerability. Our patient had stage II CRC with three high-risk features (vascular and perineural invasion, T4 tumor) and was over 75 years old, limiting her tolerance for standard platinum-based chemotherapy. Current guidelines recommend avoiding platinum escalation in this context, and single-agent fluoropyrimidine provides limited benefit in MSI-H CRC (9). Evidence supporting the use of immune checkpoint inhibitors (ICIs) as adjuvant therapy for colorectal cancer (CRC) is currently limited. A recent multi-center retrospective study involving dMMR/MSI-H CRC and gastric cancer reported a 3-year disease-free survival (DFS) rate of 94% among CRC patients who received adjuvant ICI therapy after a median follow-up of 35.5 months (22). However, the long-term efficacy and safety of adjuvant immunotherapy remain to be confirmed, and ongoing trials such as the ATOMIC study (NCT02912559) are further exploring the role of PD-1 inhibitors in stage II/III MSI-H CRC. Notably, current studies lack data on HAC, a rare histological subtype of CRC, and its treatment outcomes following adjuvant immunotherapy remain largely unknown. Age-related immunosenescence may impair T cell function and ICI responsiveness (23); however, clinical data indicate that PD-1/PD-L1 blockade is generally effective and safe in elderly patients, comparable to younger cohorts (24). In this case, eight cycles of envafolimab were well tolerated, with no grade ≥2 immune-related adverse events, suggesting feasibility of adjuvant immunotherapy in high-risk elderly MSI-H/dMMR CRC.

Limitations of this report include its single-case nature, absence of detailed tumor microenvironment profiling (e.g., PD-L1 expression, TIL density, immune cell phenotyping), and lack of long-term follow-up data to fully assess late toxicity or sustained benefit. Optimal dosing, duration, and patient selection for adjuvant immunotherapy in stage II MSI-H CRC remain undefined, with most evidence extrapolated from metastatic settings (4). Multi-center prospective studies with larger cohorts are warranted to validate the efficacy and safety of PD-1 blockade in rare CRC subtypes such as HAC, and to refine predictive biomarkers, treatment duration, and dosing strategies for personalized therapy.

## Conclusion

We report a rare case of sporadic MSI-H hepatoid adenocarcinoma of the colon in an elderly patient who achieved prolonged disease-free survival following adjuvant pembrolizumab, without experiencing any grade ≥2 immune-related adverse events. This case highlights that MSI-H/dMMR status, beyond serving as a predictive biomarker for immunotherapy in metastatic CRC, may also offer a safe and effective adjuvant treatment option for high-risk elderly patients with stage II disease. Future multi-center, prospective studies with larger cohorts are needed to validate the long-term efficacy and safety of immunotherapy in rare CRC subtypes and elderly populations, and to refine optimal dosing, treatment duration, and predictive biomarker strategies, thereby advancing precision medicine in colorectal cancer management.

## Data Availability

The original contributions presented in the study are included in the article/supplementary material. Further inquiries can be directed to the corresponding author.
